# Association of various RAS codon mutations and prognostic outcomes of patients with colorectal liver metastases after hepatectomy

**DOI:** 10.1002/cam4.70168

**Published:** 2024-10-08

**Authors:** Xiao‐Gang Wu, Wei Liu, Yan‐Yan Wang, Kun Wang, Bao‐Cai Xing

**Affiliations:** ^1^ State Key Laboratory of Holistic Integrative Management of Gastrointestinal Cancers, Beijing Key Laboratory of Carcinogenesis and Translational Research, Hepatopancreatobiliary Surgery Department I Peking University Cancer Hospital & Institute Beijing China

**Keywords:** colorectal cancer, hepatectomy, liver metastasis, prognosis, RAS codon mutation

## Abstract

**Purpose:**

The prognostic and predictive value of RAS mutations in patients with colorectal liver metastases (CRLM) who have undergone hepatectomy holds substantial importance. The present study aimed to investigate the impact of different RAS codon mutations on long‐term survival in CRLM patients.

**Methods:**

A retrospective analysis was conducted on clinicopathological data from 399 CRLM patients with RAS mutations who underwent hepatectomy between January 2000 and December 2020. The RAS mutation gene status was assessed in KRAS codons (G12, G13, Q61, and A146) and NRAS codons (G12, G13, and Q61). Survival curves were generated using the Kaplan–Meier plotter and compared using the log‐rank test. Univariate and multivariate analyses were performed to analyze the clinicopathological data.

**Results:**

In the entire cohort, patients with KRAS G12 mutations exhibited the most favorable prognosis (*p* = 0.018). Comparatively, patients harboring KRAS Q61 mutations experienced poorer overall survival (OS) with a median of 15 months versus 33 months (*p* = 0.011) when compared to those with KRAS G12 mutations. Moreover, patients with NRAS Q61 mutations also showed decreased OS with a median of 26 months versus 33 months (*p* = 0.020) in comparison to KRAS G12 mutation patients. The results of multivariate analysis showed that both KRAS Q61 mutation (HR 2.130; 95% CI 1.088–4.168; *p* = 0.027) and NRAS Q61 mutation (HR 2.877; 95% CI 1.398–5.922; *p* = 0.004) were independent influencing factors of OS. Based on all identified risk factors, patients with RAS mutation were divided into high‐risk and low‐risk groups. Notably, in the high‐risk group, the incorporation of postoperative chemotherapy was associated with longer OS, while it did not improve the survival of patients in the low‐risk group.

**Conclusions:**

KRAS Q61 and NRAS Q61 mutations are promising predictors for OS in CRLM patients after hepatectomy. Postoperative chemotherapy may significantly benefit CRLM patients with RAS mutations, particularly those identified as high‐risk.

## 1 INTRODUCTION

Colorectal cancer (CRC) ranks as the third most prevalent malignancy globally and stands as the second leading cause of cancer‐related mortality.^1^ Colorectal liver metastases (CRLM) occur in nearly half of all colorectal cancer patients, contributing to over 50% of CRC‐related deaths.^2^, ^3^ Hepatic resection is the most effective treatment option for CRLM patients.^4^, ^5^, ^6^ Despite surgical intervention, recurrence rates ranging from 60% to 80% have been observed, with the majority of recurrences transpiring within 2 years.^7^ Several clinicopathological factors, encompassing tumor size and number, synchronous and metachronous metastases, preoperative carcinoembryonic antigen (CEA) levels, and response to preoperative chemotherapy, have been employed to predict the prognosis of CRLM patients.^8^, ^9^ Recently, there has been heightened attention towards genetic and molecular markers as prognostic indicators for long‐term outcomes in CRLM patients following hepatectomy.^10^, ^11^, ^12^

The rat sarcoma viral oncogene homolog (RAS) oncogenes (KRAS, NRAS, HRAS) encode a family of GTP‐regulated switches that are recurrently mutated in human cancer.^10^ RAS mutations play a pivotal role in both the pathogenesis and prognosis of patients afflicted with CRLM.^11^, ^12^ Among these, KRAS mutations occur predominantly in approximately 30% of CRLM patients.^13^ Patients possessing KRAS mutation have been associated with worse overall survival, as KRAS‐mutated cells tend to perform increased motility, invasiveness, and metastatic potential.^14^, ^15^, ^16^, ^17^ While NRAS mutations occur less frequently compared to KRAS mutations, they are also linked to adverse prognosis and a reduced response to anti‐EGFR therapies, resembling the scenario with KRAS mutations.^10^, ^18^, ^19^

In CRLM patients, mutations in KRAS codons G12, G13, Q61, A146, and NRAS codons G12, G13, and Q61 have been detected.^20^, ^21^, ^22^ Traditionally, tumor gene mutations were regarded as general biomarkers.^11^, ^12^ However, emerging evidences indicates that different RAS codon mutations exhibit distinct biological characteristics associated with overall survival and recurrence.^21^, ^23^, ^24^, ^25^, ^26^ The prognostic and predictive value of different RAS codons on the survival of CRLM patients after hepatectomy remains uncertain. Therefore, the present study aimed to investigate the prognostic implications of different RAS codon mutations in CRLM patients undergoing liver resection.

## 2 MATERIALS AND METHODS

### 2.1 Patient selection

At the Hepatopancreatobiliary Surgery Department I of Peking University Cancer Hospital, patients who were diagnosed as CRLM pathologically after hepatectomy from January 2000 to December 2020 were included. Patients were excluded in the case of the following: no available results of RAS mutation, hepatectomy owing to recurrence after previous hepatectomy or radiofrequency ablation (RFA), with the unresectable primary tumor, staging hepatectomy, palliative hepatectomy (R2). The clinical and pathological data were collected when patients were diagnosed CRLM initially, including imaging examinations and peripheral blood tests. Tumor stage was based on the pathological reports of the primary tumor. Most patients' genomic DNA came from the specimen of primary tumor, and a small group of patients' genomic DNA was isolated from CRLM tissue specimens. All the hematoxylin and eosin‐stained tumor sections were examined. In the paraffin‐embedded tumors, the optimum tissue block was chosen for RAS mutation testing. The genomic DNA was extracted and the status of KRAS (Codons G12, G13, Q61, and A146) and NRAS (Codons G12, G13, and Q61) were determined by the Sanger sequencing or the Next‐generation sequencing (NGS). Overall survival (OS) and recurrence‐free survival (RFS) were defined as the interval between the date of liver resection and the date of death or recurrence, respectively. Informed consent was obtained from all patients, and the study was ratified by the Ethics Committee of Peking University Cancer Hospital.

### 2.2 Disease management

We discuss the application of specific treatment strategies for CRLM patient at the multidisciplinary meeting every week. A subset of patients received neoadjuvant chemotherapy or conversion therapy, and nearly half of the patients also received targeted therapy (bevacizumab). In addition, there were also patients who underwent direct surgery without chemotherapy and received adjuvant chemotherapy after hepatectomy. Before operation, abdominal‐pelvic enhanced computed tomography (CT) and magnetic resonance imaging (MRI) of liver were performed to evaluate the possibility of surgical resection. All the CRLM patients underwent hepatectomy for therapeutic purposes and achieved complete resection (R0) while saving more functional liver parenchyma if at all possible. The remaining volume of normal liver parenchyma was required to be greater than 30%, and for patients with chemotherapy‐induced liver injury, the remnant volume was preserved at over 40%. Routine intraoperative ultrasound was performed to detect occult and vanishing lesions, and surgical resection combined with RFA enhance the potential for radical surgery and reserved a greater volume of remnant liver. If preoperative chemotherapy was effective, the same chemotherapy regimen was usually adopted after liver resection. Surgical intervention was considered for recurrent cases when the overall treatment strategy was deemed potentially curative. In addition, RFA and radiotherapy were also used for local treatment of recurrent patients.

### 2.3 Follow‐up

Follow‐up duration was defined as the period between the date of liver resection and either the date of the last follow‐up or death. After the operation, patients were reviewed every 3 months for the first 2 years, every 6 months in the next 3 years, and annually thereafter. CT/MRI, liver function and tumor markers were examined to detect for tumor recurrence every time. All patients were followed up via telephone or outpatient service every 6 months after liver resection.

### 2.4 Statistical analysis

Continuous variables with a normal distribution were presented as means with standard deviation (SD) and compared using the Student *t*‐test. Continuous variables which were in conformity to normal distribution were presented as medians with interquartile range (IQR) and compared using the Mann–Whitney *U* test. Categorical variables were expressed as percentage (%) and compared using Pearson's chi‐square test or Fisher's exact test. Survival curves were generated using the Kaplan–Meier plotter, and differences were assessed using the log‐rank test. Variables showing significance in the univariate analysis were included in the multivariate analysis. SPSS 25.0 software (IBM Corp.) and R, vision 4.1.0 (www.r‐project.org) were used for statistical analysis. GraphPad Prism 8.0.1 (GraphPad Software, USA) was used for Kaplan–Meier survival curves. In all analyses, differences were considered statistically significant only if *p* < 0.050.

## 3 RESULTS

**FIGURE 1:**
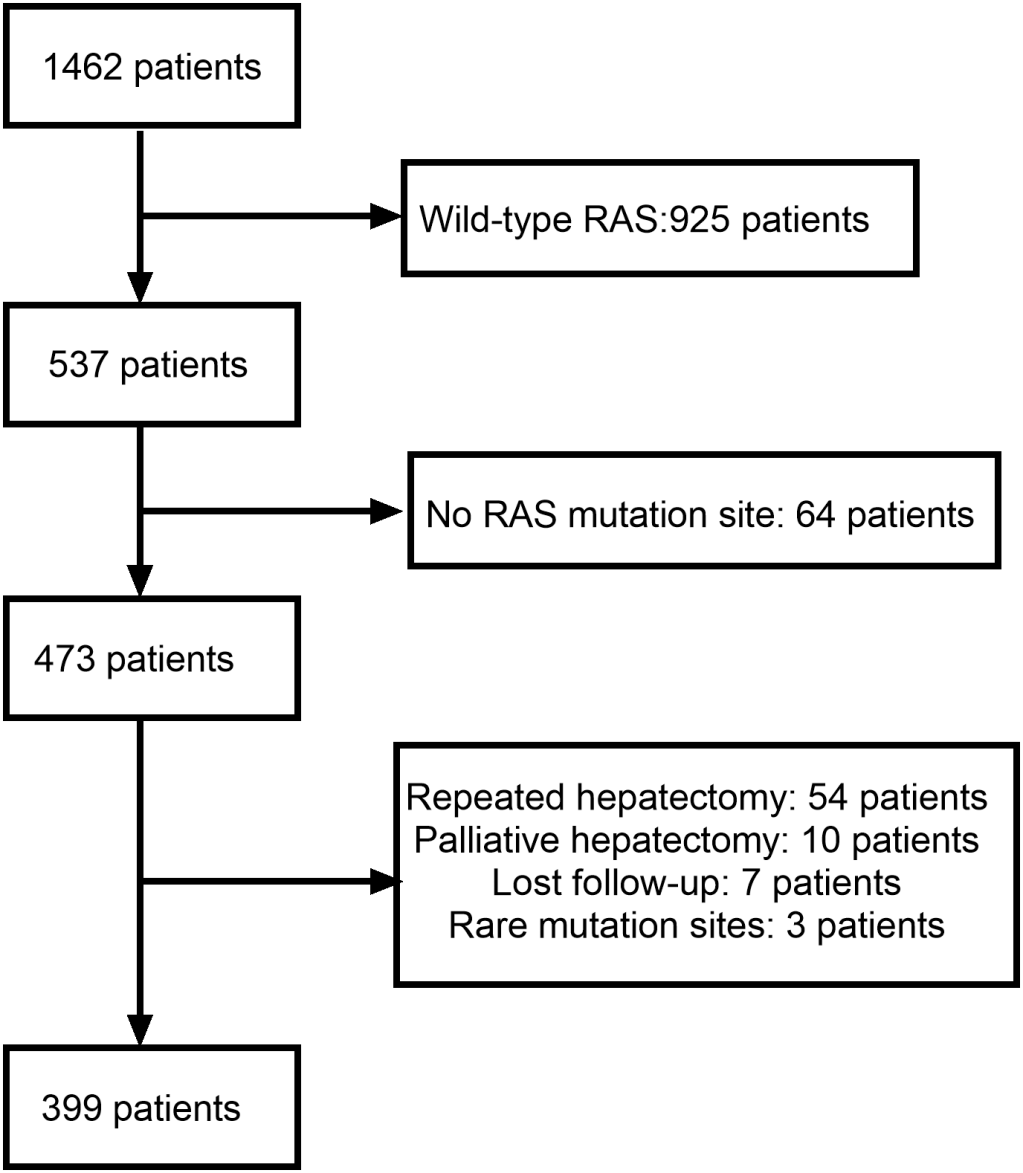
Flowchart of inclusion and exclusion criteria.

From January 2000 to December 2020, a total of 1462 consecutive CRLM patients who underwent hepatectomy were enrolled at the Hepatopancreatobiliary Surgery Department I of Peking University Cancer Hospital. There were 937 patients with wild‐type RAS gene, and 537 patients with RAS mutations, including 492 cases with KRAS mutations, 44 cases with NRAS mutations, and 1 patient with HRAS mutation. A total of 138 patients were excluded based on the criteria, including 64 patients who had unknown RAS mutation sites, 54 patients who underwent hepatectomy due to recurrence after liver surgery or RFA, 10 patients who underwent palliative hepatectomy, 7 patients who were lost to follow‐up, and 3 patients had KRAS A59, KRAS K117, and HRAS mutation respectively. Ultimately, a total of 399 patients who met the predefined inclusion criteria were included in the study (Figure 1). The majority of these patients carried KRAS G12 mutations (*n* = 260, 65.2%), followed by mutations in KRAS G13 (*n* = 82, 20.6%), KRAS Q61 (*n* = 12, 3.0%), KRAS A146 (*n* = 14, 3.5%), NRAS G12 (n = 12, 3.0%), and NRAS G13 (*n* = 4, 1.0%) and NRAS Q61 (*n* = 15, 3.8%). And there were no patients who had co‐mutation of KRAS and NRAS codons.

### 3.1 Clinicopathological characteristics

A total of 399 patients met the inclusion criteria and were included in the study. KRAS mutations were detected in 368 patients (92.2%), and NRAS mutations were detected in 31 patients (7.8%). Baseline clinicopathological characteristics of the population have been succinctly summarized in Table 1 and patients were stratified by KRAS and NRAS mutation. Compared with NRAS‐mutation patients, KRAS‐mutation patients with right‐side primary tumors were more common (29.1% vs. 9.7%; *p* = 0.02). No other differences were found between patients with KRAS and NRAS mutations.

**TABLE 1:** Clinical characteristics of patients with KRAS and NRAS mutation.

Characteristic	All patients (*n* = 399)	Mutant KRAS (*n* = 368)	Mutant NRAS (*n* = 31)	*p* value
Age, years, median (IQR)	58 (51–64)	58 (50–63)	63 (54–65)	0.108^a^
Gender				0.765^b^
Male	229 (57.4)	212 (57.6)	17 (54.8)	
Female	170 (42.6)	156 (42.4)	14 (45.2)	
Primary tumor location				0.020^b^
Left side	289 (72.4)	261 (70.9)	28 (90.3)	
Right side	110 (27.6)	107 (29.1)	3 (9.7)	
Primary T stage				0.723^b^
T1–2	31 (7.8)	28 (7.6)	3 (9.7)	
T3–4	368 (92.2)	340 (92.4)	28 (90.3)	
Primary N stage				0.287^b^
N0	124 (31.1)	117 (31.8)	7 (22.6)	
N1–2	275 (68.9)	251 (68.2)	24 (77.4)	
Synchronous CRLM				0.214^b^
Yes	255 (63.9)	232 (63.0)	23 (74.2)	
No	144 (36.1)	136 (37.0)	8 (25.8)	
No. of CRLM, median (IQR)	3 (1–5)	2 (1–5)	3 (1–7)	0.369^a^
Largest diameter, mm, median (IQR)	29 (19–43)	29.5 (19.25–43)	25 (17–42)	0.588^a^
Distribution				0.958^b^
Unilobar	182 (45.6)	168 (45.7)	14 (45.2)	
Bilobar	217 (54.4)	200 (54.3)	17 (54.8)	
Extrahepatic disease				0.158^b^
Yes	77 (19.3)	74 (20.1)	3 (9.7)	
No	322 (80.7)	294 (79.9)	28 (90.3)	
Preoperative CEA, ng/ml, median (IQR)	17.59 (6.24–74.91)	16.59 (6.23–72.82)	27.35 (7.95–139.96)	0.516^a^
Preoperative CA19‐9, kU/L, median (IQR)	42.5 (16.14–177.9)	45.48 (16.17–199.48)	27.3 (12.43–61.03)	0.152^a^
Preoperative chemotherapy				0.714^b^
Yes	319 (79.9)	295 (80.2)	24 (77.4)	
No	80 (20.1)	73 (19.8)	7 (22.6)	
Postoperative chemotherapy				0.783^b^
Yes	279 (69.9)	258 (70.1)	21 (67.7)	
No	120 (30.1)	110 (29.9)	10 (32.3)	
Combination bevacizumab				0.438^b^
Yes	194 (48.6)	181 (49.2)	13 (41.9)	
No	205 (51.4)	187 (50.8)	18 (58.1)	
Surgical margin				0.458^b^
R0	334 (83.7)	307 (83.4)	27 (87.1)	
R1	65 (16.3)	61 (16.6)	4 (12.9)	

*Note*: Data were shown as *n* (%) unless otherwise specified.

Abbreviations: CA19‐9, carbohydrate antigen 19–9; CEA, carcinoembryonic antigen; CRLM, colorectal liver metastases; IQR, interquartile range; RFA, radiofrequency ablation; R0, tumor cells free margin ≥1 mm; R1, the existence of tumor cells <1 mm of the resection margin.

^a^ Data comparison between two groups using the student's *t*‐test.

^b^ Data comparison between two groups using the Chi square test.

### 3.2 Survival outcome

**FIGURE 2:**
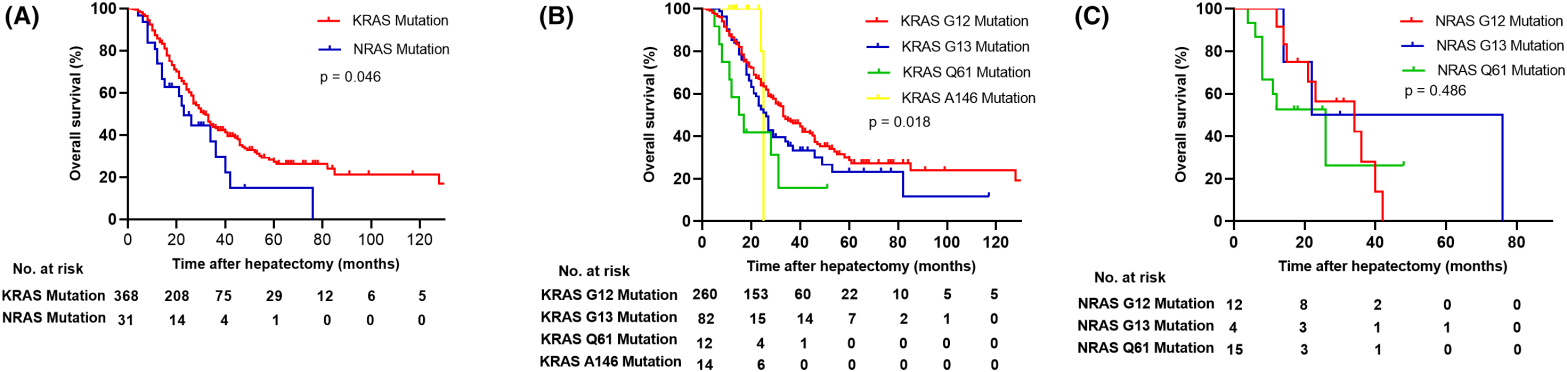
Overall survival in patients with colorectal liver metastasis stratified by KRAS and NRAS mutations (A), various KRAS codon mutations (B), and various NRAS codon mutations (C) after liver resection.

**FIGURE 3:**
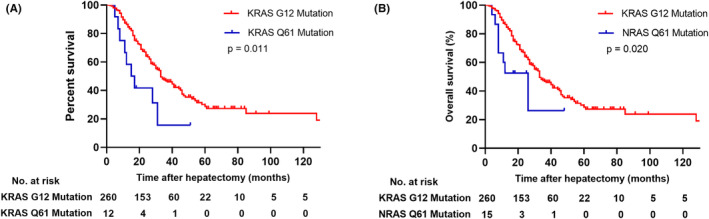
Overall survival in patients with colorectal liver metastasis stratified by KRAS G12 and KRAS Q61 mutations (A), KRAS G12 and NRAS Q61 mutations (B) after liver resection.

Survival outcomes were thoroughly evaluated in the study, and the median follow‐up time was 38 months. In the entire cohort, 297 patients (74.4%) experienced recurrence, while 183 patients (45.9%) still survived at the end of the follow‐up period. The median RFS for the total patients was 9 months. The 1‐, 3‐, and 5‐year RFS rates were 37.0%, 25.4%, and 20.7%, respectively. In KRAS codons, the median RFS were 9 months, 7 months, 3 months, and 21 months for patients with G12 mutations, G13 mutations, Q61 mutations, and A146 mutations respectively. In NRAS codons, the median RFS were 4 months, 6 months, and 4 months for patients with G12 mutations, G13 mutations, and NRAS Q61 mutations respectively. There was no statistical difference in the median RFS among all codons (*p* = 0.089). The median OS for the total patients was 31 months. The 1‐, 3‐, and 5‐year OS rates were 95.5%, 53.0%, and 25.6%, respectively. Compared to NRAS mutation patients, patients with KRAS mutations had better survival. More specifically, the median OS of patients with KRAS mutations was 32 months, whereas the median OS was 23 months for NRAS mutations (*p* = 0.046; Figure 2A). In KRAS codons, the median OS were 33 months, 26 months, 15 months, and 25 months for patients with G12 mutations, G13 mutations, Q61 mutations, and A146 mutations respectively (*p* = 0.018; Figure 2B). The difference of OS between KRAS G12 and G13 did not meet statistical standards. Although the survival curve of KRAS G13 and Q61 did not overlap, there was no statistical significance. Due to insufficient follow‐up time, the survival curve of KRAS A146 crossed with other codons. Among NRAS codons, the median OS were 34 months, 22 months, and 26 months for patients with G12 mutations, G13 mutations, and Q61 mutations respectively (*p* = 0.486; Figure 2C). The survival curves overlapped and intersected with each other. Although the median OS for patients with NRAS G12 mutations was longer than other NRAS codons, there was no statistical significance. Among all RAS codons, KRAS G12 mutation patients in the cohort had a superior prognosis. In addition, KRAS and NRAS Q61 mutation patients had worse statistical survival. Compared to KRAS G12 mutation patients, KRAS Q61 mutation patients had worse OS (*p* = 0.011; Figure 3A), similar to NRAS Q61 mutation patients (*p* = 0.020; Figure 3B).

Univariate analysis revealed that KRAS G12, KRAS/NRAS Q61 mutations were significantly associated with OS for different codons. In addition, primary N stage, number of CRLM, extrahepatic disease, preoperative CA19‐9, postoperative chemotherapy, and combination of targeted therapy were associated with OS in univariate analysis (all *p* < 0.05). All variables (*p* < 0.05) were included in a subsequent multivariate Cox regression analysis. In multivariable analysis, KRAS Q61 mutation (HR 2.130; 95% CI 1.088–4.168; *p* = 0.027), NRAS Q61 mutation (HR 2.877; 95% CI 1.398–5.922; *p* = 0.004), primary N stage (HR:1.408; 95% CI 1.041–1.905; *p* = 0.026), number of CRLM (HR:1.616; 95% CI 1.217–2.337; *p* = 0.002), extrahepatic disease (HR:1.686; 95% CI 1.177–2.262; *p* = 0.002), and postoperative chemotherapy (HR:0.703; 95% CI 0.531–0.929; *p* = 0.013) were independent risk factors for OS. Univariate and multivariate analyses for OS risk factors are shown in Table 2.

**TABLE 2:** Univariate and multivariate analyses to identify risks of overall survival.

Prognostic factor	Univariable *p* value	Multivariable analysis HR (95% CI)	Multivariable *p* value
RAS codon			
KRAS G12 mutation (no/yes)	0.018	0.838 (0.620–1.132)	0.248
KRAS Q61 mutation (no/yes)	0.022	2.130 (1.088–4.168)	0.027
NRAS Q61 mutation (no/yes)	0.027	2.877 (1.398–5.922)	0.004
KRAS G13 mutation (no/yes)	0.271		
KRAS A146 mutation (no/yes)	0.174		
NRAS G12 mutation (no/yes)	0.337		
NRAS G13 mutation (no/yes)	0.855		
Age, years (<60/≥60)	0.477		
Gender (male/female)	0.077		
Primary tumor location (left/right)	0.448		
Primary T stage (T1–2/ T3–4)	0.156		
Primary N stage (N0/N1–2)	0.014	1.408 (1.041–1.905)	0.026
Synchronous CRLM (no/yes)	0.467		
No. of CRLM (single/multiple)	0.005	1.616 (1.197–2.182)	0.002
Largest diameter (<50 mm/≥50 mm)	0.322		
Distribution (unilobar/bilobar)	0.116		
Extrahepatic disease (no/yes)	0.003	1.686 (1.217–2.337)	0.002
Preoperative CEA (<200 ng/mL/≥200 ng/mL)	0.082		
Preoperative CA19‐9 (<37 kU/L/≥37 kU/L)	0.023	1.297 (0.977–1.721)	0.072
Preoperative chemotherapy (no/yes)	0.660		
Postoperative chemotherapy (no/yes)	0.014	0.703 (0.531–0.929)	0.013
Combination bevacizumab (no/yes)	0.045	1.190 (0.893–1.587)	0.235
Surgical margin (R0/R1)	0.764		

Abbreviations: CA19‐9, carbohydrate antigen 19–9; CEA, carcinoembryonic antigen; CI, confidence interval; CRLM, colorectal liver metastases; HR, hazard ratio; RFA, radiofrequency ablation; R0, tumor cells free margin ≥1 mm; R1, the existence of tumor cells <1 mm of the resection margin.

### 3.3 Tumor prognostic scoring system

**FIGURE 4:**
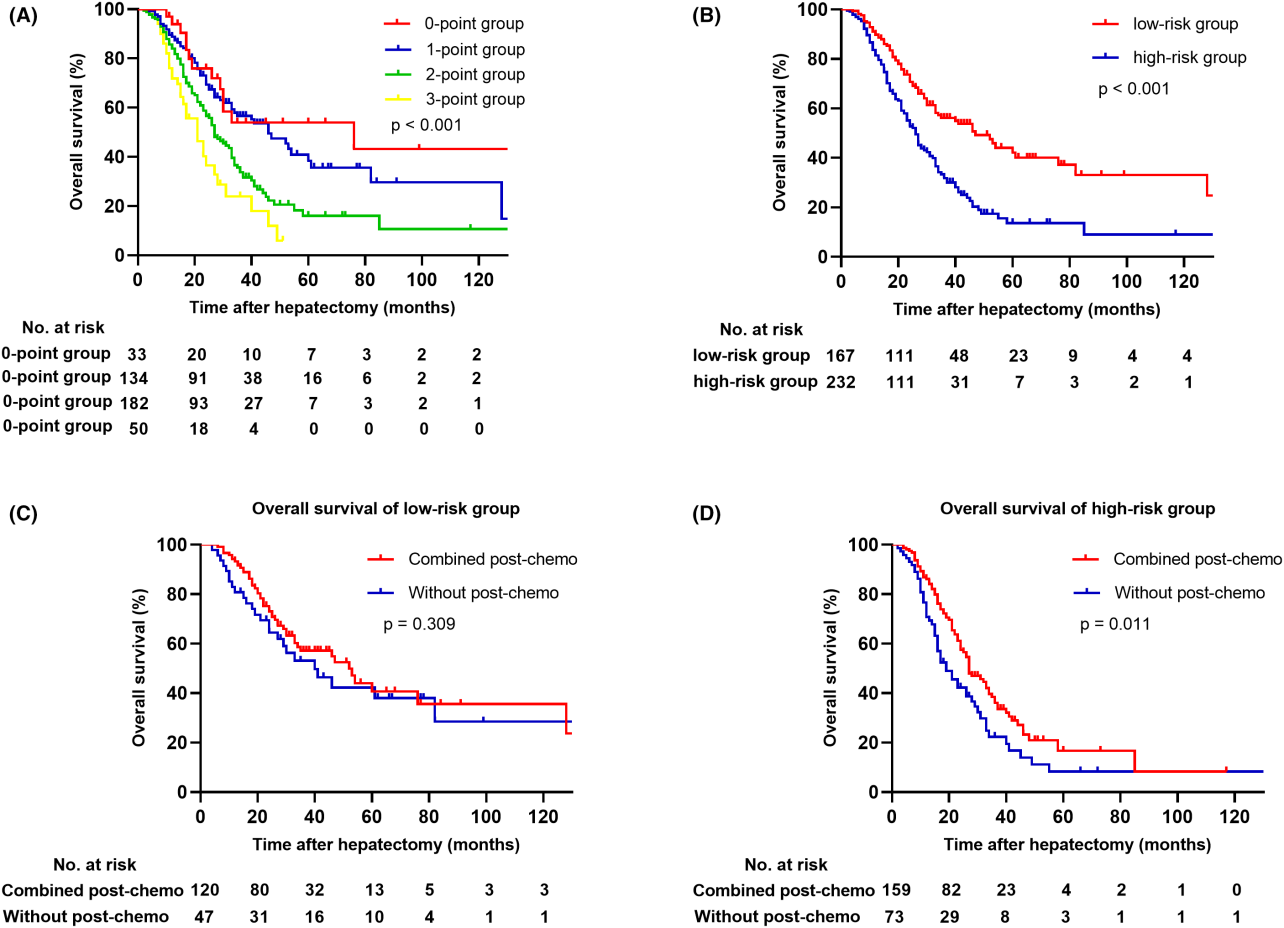
Overall survival of patients with different risk points according to the tumor prognostic scoring system respectively (A); Overall survival of patients in low‐risk and high‐risk groups (B); Overall survival of patients in low‐risk group (C) and high‐risk group (D) stratified by postoperative chemotherapy.

**FIGURE 5:**
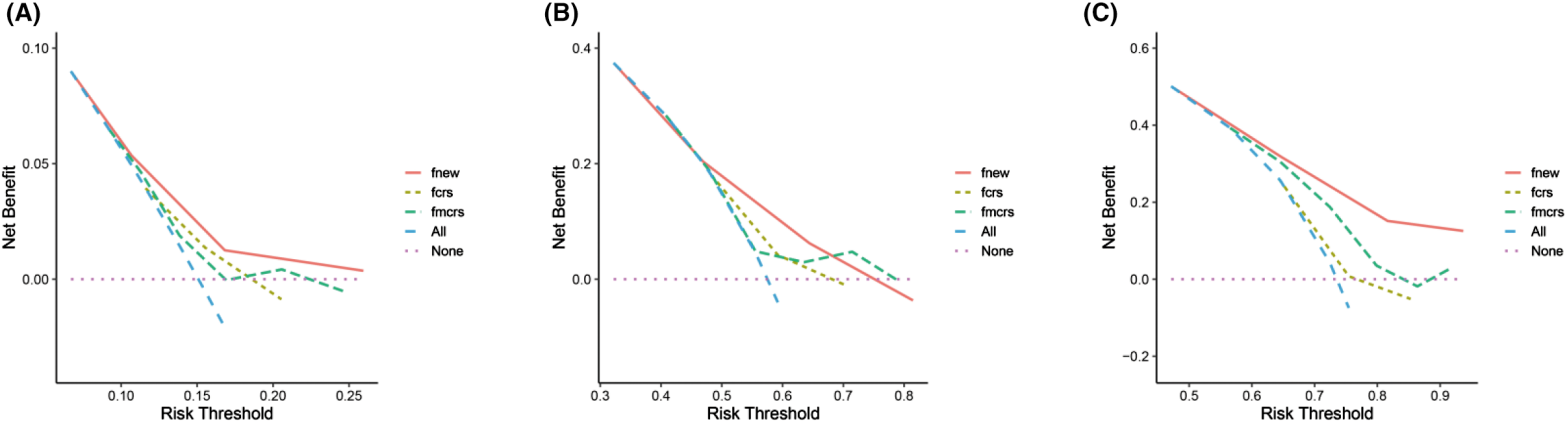
DCA curves showed the performance of the new scoring system, the CRS, and the m‐CRS in predicting overall survival rate of 1‐year (A), 3‐year (B), and 5‐year (C).

According to multivariable analysis, only postoperative chemotherapy was protective factor for CRLM patients with RAS mutations. Consequently, the remaining five risk factors, including KRAS Q61 mutation, NRAS Q61 mutation, primary N stage, number of CRLM, and extrahepatic disease were chosen as criteria for a tumor prognostic scoring system. Each risk factor was assigned 1 point, and the lowest and highest scores were 0 and 3 points for the entire cohort, shown in Figure 4A, as patients did not have all the independent risk factors at the same time in the cohort. According to the scoring system, patients were categorized into the low‐risk group (0–1 point; *n* = 167) and the high‐risk group (2–3 points; *n* = 232). The median OS in the 2 groups was 47 months and 26 months, respectively (*p* < 0.001, Figure 4B). The high‐risk group patients had lower 1‐, 3‐, and 5‐year OS rates (81.4%, 31.8%, 13.6%, respectively), compared to those of the low‐risk group (92.8%, 56.2%, 42.2%, respectively). Within the low‐risk group, there was no statistically significant difference in OS between patients who received postoperative chemotherapy and those who did not (52 months vs. 40 months; *p* = 0.309; Figure 4C). Conversely, within the high‐risk group, postoperative chemotherapy demonstrated a statistically significant improvement in OS. Patients who received postoperative chemotherapy had a median OS of 27 months, whereas those without chemotherapy had a median OS of 19 months (*p* = 0.011; Figure 4D). In order to demonstrate the efficacy of our new tumor prognostic scoring system and compare the superiority with the CRS and the m‐CRS, we introduced the decision curve analysis (DCA). DCA curves illustrated our new tumor prognostic scoring system possessed clinical net benefits in predicting the 1‐, 3‐, and 5‐year OS rates, compared with the CRS and the m‐CRS (Figure 5).

## 4 DISCUSSION

Currently, RAS has emerged as one of the most frequently utilized biomarkers for predicting the survival outcomes of CRLM patients after surgical resection.^11^ It has been established that patients harboring RAS mutations tend to have worse prognosis and higher likelihood of recurrence.^11^, ^12^ However, the prognostic impacts of specific RAS codon mutations have not been well recognized.^24^, ^25^, ^26^ This study aimed to investigate the impact of distinct RAS codon mutations on RFS and OS of CRLM patients who underwent curative hepatectomy. Additionally, a prognostic scoring system for tumor prognosis was developed for patients with RAS mutations, considering independent risk factors identified through multivariable analysis for OS. The results of this investigation revealed varying prognoses among patients with specific RAS codon mutations. Notably, patients with the KRAS G12 mutation exhibited a more favorable prognosis, whereas those with KRAS and NRAS Q61 mutations experienced worse OS. Multivariable analysis indicated that six variables, including KRAS Q61 mutation, NRAS Q61 mutation, primary N stage, number of CRLM, extrahepatic disease, and receipt of postoperative chemotherapy, were independent risk factors for patients with RAS mutations. Furthermore, subgroup analysis provided insightful evidence that longer OS was associated with postoperative chemotherapy in high‐risk patients, as determined by the tumor prognostic scoring system.

The present study identified specific RAS codon mutations in 399 patients. There were 368 patients with specific KRAS codon mutations included in this study and the majority were KRAS G12 (70.7%) and G13 (22.3%), while A146 and Q61 were less common, identified in less than 10% of the patients with specific KRAS codon mutations. In line with previously published data, specific NRAS codon mutations were identified in 31 patients, with NRAS Q61 mutations being the most frequent (48.4%), followed by G12 mutations (38.7%), and G13 mutations (12.9%).^10^, ^18^ Compared with KRAS codon mutations, the proportion of NRAS codon mutations was different as the number of patients with NRAS mutations was small. Regarding clinicopathological characteristics, it was observed that patients with mutant KRAS had a higher prevalence of right‐side primary tumors, consistent with findings from a prior study of Cercek.^10^ In their retrospective evaluation of 2764 metastatic colorectal cancer (mCRC) patients, Cercek et al. reported that 3% of mCRC patients possessed NRAS mutations, which were more commonly observed in left‐side primary tumors.^10^

Among the various codons analyzed, the RFS of patients with KRAS A146 mutations was longer than other codons, although statistical significance was not reached (*p* = 0.089). However, it's noteworthy that the number of patients with KRAS A146 was relatively small and some studies obtained different conclusions for specific categories of CRLM patients. For instance, Saadat et al. conducted a comprehensive evaluation of 938 patients with mCRC following hepatectomy and found that patients with exon 4, including KRAS A146 mutations, exhibited a more favorable prognosis.^26^ However, in a multicenter prospective trial, Erve et al. analyzed 419 CRC patients with unresectable liver metastasis and demonstrated that mCRC patients with KRAS A146 mutations had a higher tumor burden and worse prognosis.^21^ In our study, univariate analysis further revealed a significant association between KRAS G12 mutations and OS. Most RAS mutations were KRAS G12 and the median OS was 33 months. Previous studies pointed out that KRAS G12 mutation was an independent risk factor for CRLM patients, often correlated with a shorter OS compared with wild‐type KRAS patients.^24^, ^25^ In the present study, patients with KRAS G12 mutations had a more favorable prognosis than those with KRAS Q61 or NRAS Q61 mutations. Although the number of cases with KRAS Q61 and NRAS Q61 mutations was limited, the difference was statistically significant. Similarly, Guo et al. discovered that KRAS exon 3 mutations, including KRAS Q61 mutations, predicted a worse prognosis in 1816 CRC patients.^27^ Moreover, in the study conducted by Cercek, the presence of an NRAS mutation was a more aggressive marker in mCRC patients, linked with poor survival and worse outcomes than both mutant‐type and wild‐type KRAS mCRC.^10^ Few studies further discussed the survival differences between different codons in NRAS mutations.

Multivariable analysis in this study identified six variables as independent risk factors that independently influenced the OS of CRLM patients with RAS mutation. From these variables, we selected five clinicopathological characteristics as criteria for the tumor prognostic scoring system, including KRAS Q61 mutation, NRAS Q61 mutation, primary N stage, number of CRLM, and extrahepatic disease. According to the scoring system, the survival outcome of patients in 2‐, and 3‐point patients were significantly different from 0‐, and 1‐point patients. Hence, we can treat the scoring system as a reliable model to predict the survival and prognosis of CRLM patients with RAS mutation after liver resection. Several prognostic models were developed to predict recurrence and survival in CRLM patients. These models typically incorporated both clinicopathological factors and genetic molecular markers. The clinical risk score (CRS) was the most widely‐used score described by Fong et al.^8^ Patients with high CRS had worse OS rates than those with low CRS, and perioperative chemotherapy was only associated with a survival advantage in high CRS patients.^8^ However, the validity of CRS was questioned in the era of modern chemotherapy and targeted therapy.^12^, ^28^, ^29^ Brudvik et al. retrospectively analyzed 564 CRLM patients and developed the modified clinical score (m‐CRS). They found that the m‐CRS outperformed the CRS and provided a quick preoperative assessment of the expected survival benefit.^12^ In addition, Margonis et al. developed the genetic and morphological evaluation (GAME) score by combing clinicopathological and clinically available biological indicators, including KRAS mutation, tumor burden score (TBS), redefined CEA level (20 ng/mL), and extrahepatic disease.^28^ Sasaki et al. have demonstrated strong calibration of the GAME score and higher discrimination than the CRS and m‐CRS.^29^ Drawing from the results of this study, it is evident that CRLM patients with distinct RAS codon mutations did not share identical prognosis. Consequently, our tumor prognostic scoring system proposed herein offers a valuable tool for predicting the survival and prognosis among patients with diverse RAS codon mutations.

For patients with unresectable initially CRLM, conversion chemotherapy allowed 12.5% of patients to be rescued by liver resection.^30^ Secondary resections and extrahepatic resections after conversion chemotherapy improved long‐term survival for these patients.^30^ In addition, neoadjuvant chemotherapy was associated with a better prognosis for patients with resectable disease, which selected those patients who could benefit from liver resection.^31^ Based on our proposed tumor prognostic scoring system, CRLM patients with RAS mutation were divided into high‐risk and low‐risk groups. Within the high‐risk group, patients who received postoperative chemotherapy exhibited statistically significant improvements in survival compared to those who did not undergo postoperative chemotherapy. Nevertheless, there was no statistical difference in the low‐risk group. Adam et al. analyzed a multicentric cohort of 1471 patients resected for solitary, metachronous, primarily resectable CRLMs without the extrahepatic disease. They found postoperative chemotherapy was associated with better OS and DFS when the tumor diameter exceeds 5 cm.^32^ A meta‐analysis of randomized trials revealed that no OS benefit was documented from the use of postoperative chemotherapy with single‐agent fluoropyrimidines. But significant disease‐free survival benefit with the use of postoperative chemotherapy was observed.^33^ Combined with our findings, postoperative chemotherapy may significantly benefit CRLM patients with RAS mutations, especially those categorized as high‐risk.

Nevertheless, several limitations must be considered when interpreting these results. First, as a retrospective study, there may be biases related to patient selection, missing data, and follow‐up. Second, all the genetic tests for CRLM patients were not completed at the same laboratory. Additionally, RAS mutations were detected in primary tumors or liver metastases, which might introduce certain nuances that could influence the ultimate outcomes.^34^ Finally, our study did not count other molecular markers that significantly influence prognoses, such as microsatellite instability and human epidermal growth factor receptor‐2.^35^, ^36^

## 5 CONCLUSION

KRAS and NRAS Q61 mutations are promising predictors for OS in CRLM patients after hepatectomy. Postoperative chemotherapy may significantly benefit CRLM patients with RAS mutations, particularly those identified as high‐risk.

## AUTHOR CONTRIBUTIONS

**Xiao‐Gang Wu:** Data curation (equal); formal analysis (lead); investigation (equal); methodology (equal); software (equal); visualization (lead); writing – original draft (lead). **Wei Liu:** Data curation (equal); formal analysis (equal); investigation (equal); methodology (equal); software (equal); visualization (supporting); writing – original draft (supporting). **Yan‐Yan Wang:** Formal analysis (supporting); methodology (supporting); software (supporting); validation (supporting). **Kun Wang:** Conceptualization (equal); funding acquisition (equal); project administration (equal); supervision (equal). **Bao‐Cai Xing:** Conceptualization (equal); funding acquisition (equal); project administration (equal); resources (equal); supervision (equal).

## FUNDING INFORMATION

This study was supported by grants from the National Natural Science Foundation of China (Grant No. 81874143 and Grant No. 31971192); Beijing Hospitals Authority Mission Plan (Grant No. SML20191101).

## CONFLICT OF INTEREST STATEMENT

The authors declare no conflicts of interest.

## ETHICS APPROVAL STATEMENT

The Ethics Committee of Peking University cancer hospital approved this study.

## PATIENT CONSENT STATEMENT

All materials and data were obtained with agreement from patients and informed consent was signed.

## Data Availability

All data and material relevant to the study are available from the authors upon request.
